# Volatile Organic Compounds, Bacterial Airway Microbiome, Spirometry and Exercise Performance of Patients after Surgical Repair of Congenital Diaphragmatic Hernia

**DOI:** 10.3390/molecules26030645

**Published:** 2021-01-26

**Authors:** Gert Warncke, Georg Singer, Jana Windhaber, Lukas Schabl, Elena Friehs, Wolfram Miekisch, Peter Gierschner, Ingeborg Klymiuk, Ernst Eber, Katarina Zeder, Andreas Pfleger, Beate Obermüller, Holger Till, Christoph Castellani

**Affiliations:** 1 Department of Paediatric and Adolescent Surgery, Medical University Graz, 8036 Graz, Austria; gert.warncke@medunigraz.at (G.W.); jana.windhaber@klinikum-graz.at (J.W.); schabl.lukas@gmail.com (L.S.); elena.friehs@stud.medunigraz.at (E.F.); beate.obermueller@medunigraz.at (B.O.); holger.till@medunigraz.at (H.T.); christoph.castellani@medunigraz.at (C.C.); 2 Department of Anesthesiology and Intensive Care Medicine, Rostock Medical Breath Research Analytics and Technologies (ROMBAT), Rostock University Medical Centre, 18057 Rostock, Germany; wolfram.miekisch@uni-rostock.de (W.M.); peter.gierschner@gmx.net (P.G.); 3 Core Facility Molecular Biology, Center for Medical Research, Medical University of Graz, 8036 Graz, Austria; ingeborg.klymiuk@medunigraz.at; 4 Department of Paediatrics and Adolescent Medicine, Division of Paediatric Pulmonology and Allergology, Medical University of Graz, 8036 Graz, Austria; ernst.eber@medunigraz.at (E.E.); katarina.zeder@medunigraz.at (K.Z.); andreas.pfleger@medunigraz.at (A.P.)

**Keywords:** CDH, microbiome, VOCs, spiroergometry, outcome

## Abstract

The aim of this study was to analyze the exhaled volatile organic compounds (VOCs) profile, airway microbiome, lung function and exercise performance in congenital diaphragmatic hernia (CDH) patients compared to healthy age and sex-matched controls. A total of nine patients (median age 9 years, range 6–13 years) treated for CDH were included. Exhaled VOCs were measured by GC–MS. Airway microbiome was determined from deep induced sputum by 16S rRNA gene sequencing. Patients underwent conventional spirometry and exhausting bicycle spiroergometry. The exhaled VOC profile showed significantly higher levels of cyclohexane and significantly lower levels of acetone and 2-methylbutane in CDH patients. Microbiome analysis revealed no significant differences for alpha-diversity, beta-diversity and LefSe analysis. CDH patients had significantly lower relative abundances of *Pasteurellales* and *Pasteurellaceae*. CDH patients exhibited a significantly reduced Tiffeneau Index. Spiroergometry showed no significant differences. This is the first study to report the VOCs profile and airway microbiome in patients with CDH. Elevations of cyclohexane observed in the CDH group have also been reported in cases of lung cancer and pneumonia. CDH patients had no signs of impaired physical performance capacity, fueling controversial reports in the literature.

## 1. Introduction

Congenital diaphragmatic hernia (CDH) is a rare disease occurring with an incidence of 1:2000–1:5000 live births [1]. CDH is caused by disturbances in the formation of the diaphragm in the eighth week of gestation, leaving a defect with persistent communication between the abdominal and thoracic cavity [2]. Typically, this defect is located in the dorsal aspect of the diaphragm (Bochdalek hernia, 95% of cases, mostly located on the left side). Ventral hernias (Morgagni hernia) are rarer and typically located on the right side [3].

In fetuses with CDH, abdominal organs herniate into the thorax, subsequently restricting pulmonary development on the affected but also on the contralateral side. Consequently, patients with CDH suffer from pulmonary hypoplasia and vascular malformation with thickened muscle layers causing pulmonary hypertension with right ventricular dysfunction and left ventricular hypoplasia, reduced mobility of the diaphragm and impaired alveolar growth [4,5].

Even after surgical repair, 30–50% of CDH patients show—amongst others—persistent respiratory morbidity with impaired lung function [6,7,8] and/or recurrent respiratory infections [9]. While reduced lung function may be attributed to the congenital defect with lung hypoplasia and alveolar growth disturbances, the underlying reason for the recurrent infections may only partly be explained by impaired lung function. However, alterations of bacterial colonization can be speculated. Over recent decades, scientists have postulated sterility of the respiratory tract. However, with the advent of DNA based sequencing methods, microbial colonization of the healthy respiratory tract has been demonstrated in the last years [10]. This has led to the term “pulmonary microbiome” describing the collective genome of bacteria, archaea, fungi and viruses inhabiting the respiratory tract. Overall, there are still very limited data focusing on the pulmonary microbiome in pediatrics. While there is some evidence of alterations of the pulmonary microbiome in cases of asthma and cystic fibrosis [11,12], there are currently no data published concerning CDH patients.

While some studies describe reduced exercise tolerance in addition to impaired lung function in patients after CDH repair [5,13,14], others report normal values compared to healthy peers [7,15]. All of these studies rely on exercise performance testing, but do not look in the depth of the patients’ metabolism. The emerging field of volatile organic compounds (VOCs) analysis in patients may offer novel insights. Additionally to oxygen, nitrogen and carbon dioxide, human breath contains several hundred different VOCs [16]. Among others, the VOC profile contains carbohydrates, ketones, aldehydes, cyclic components and sulphur- or nitrogen containing compounds [16]. Some of these substances have been attributed to the metabolic and inflammatory processes of the host [17], others may also be related to the (pulmonary) microbiome.

Although the analysis of body odors, for instance the fruity smell of ketones in the breath of diabetic patients, goes back many thousand years in medical history, only recent technical developments have allowed a detailed VOC analysis. For instance, the concentration of exhaled VOC profiles differs between type I diabetes patients and healthy children [18], and metabolic adaptation through postprandial hyperglycemia and related oxidative stress is immediately reflected in exhaled breath VOC concentrations [19]. Breath VOC profiles may help to understand basic mechanisms and metabolic adaptation accompanying progression of chronic kidney disease in pediatric patients at an early stage [20]. Investigations of children with cystic fibrosis have revealed increased levels of pentane correlating to nutritional status and lung function [21]. While the pulmonary long-term sequelae of CDH have been described in several reports, examinations of exhaled VOC profiles of patients after surgical repair of CDH as potential noninvasive disease markers have not yet been published. Thus, it was the aim of this study to analyze the breath VOC profile, airway microbiome, lung function and exercise performance of patients after CDH repair compared to healthy age and sex-matched controls in order to gain more detailed information about the pathophysiology of this disease.

## 2. Results

Nine patients following surgical repair of a CDH were recruited for long-term follow-up examinations consisting of assessment of the exhaled VOC profile, airway microbiome, lung function and exercise performance. As a control group, nine age and sex-matched controls were enrolled. The median age at the examination was 9 years (IQR 5). Within each group, six patients were male and three were female. The median gestational age of the CDH patients was 39 weeks (IQR 3.8), median birth weight was 3.4 kg (IQR 0.7). CDH occurred on the left side in five, the right side in three and bilaterally in one patient. The liver was partially herniated into the thoracic cavity in two patients. CDH patients were ventilated conventionally for a median of 7.5 days (IQR 17). One patient required high frequency oscillation. Three patients were on inhalative nitric oxide because of pulmonary hypertension.

Surgical repair was performed on median day of life four (IQR 6). Eight patients underwent direct closure and one underwent a patch repair. There was no recurrence. In the post-operative medical history two of the nine CDH patients reported recurrent respiratory infections. Eight of the nine patients with CDH and all nine control patients reported feeling fit in daily life.

Not all of the patients were eligible for all examinations. Table 1 gives an overview of the data available for matched pair analysis.

### 2.1. Clinical Examination

There were no significant differences for height, body weight, BMI, muscle mass or body fat between the groups (Table 2).

### 2.2. Breath VOC Profile

In the breath samples a total of 67 different VOCs could be identified. Levels of 35 VOCs were not consistently above the limit of quantification (LOQ) and therefore had to be excluded for further quantitative analysis.

The remaining 32 substances were further processed and used for group comparison. Heatmap and dendrogram analysis showed different unspecific clusters. Alterations in the following 20 substances were significantly affected by room air contamination (levels in room air >20% of exhaled concentration): 1-methylbenzene, 1-propanol, 2,3-butandione, 2-butanone, 2-phenoxyethanol, 3-methyl-2-butanone, α-pinene, benzene, ethanol, ethylbenzene, hexanal, n-hexane, isopropylalcohol, nonanal, nonanone, octane, pentan, pentanal, p-xylene and toluene and were thus excluded as potential biomarkers (Supplementary Figure S1).

**Figure 1:**
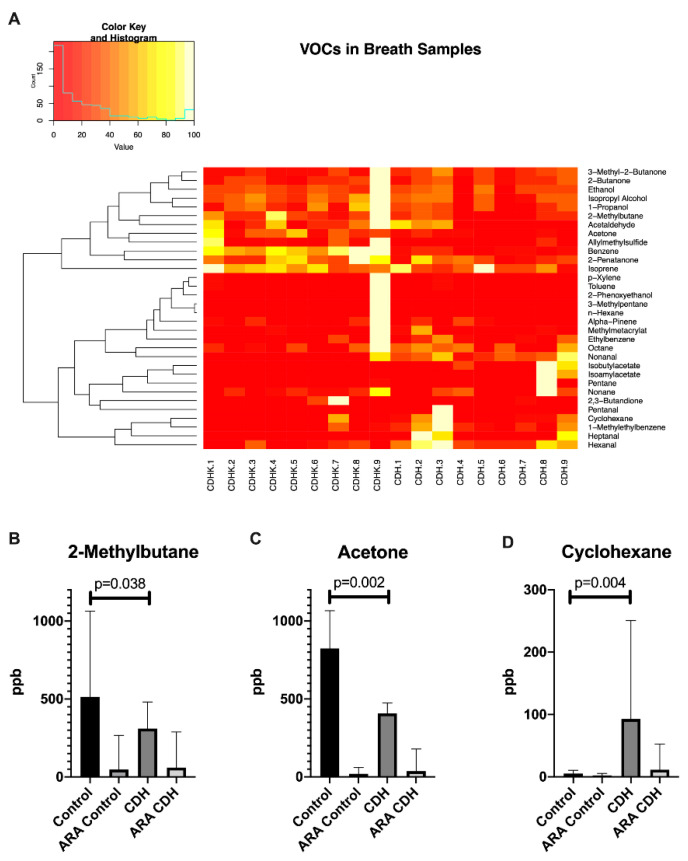
Concentrations of selected VOCs in breath samples of control patients and CDH patients (**A**). Concentrations of 2-methylbutane (**B**), acetone (**C**) and cyclohexane (**D**) were significantly different between the two groups; ARA: ambient room air.

Out of the remaining 12 substances, nine substances did not show significant differences between the groups (Supplementary Figure S2). Significant differences occurred for 2-methylbutane, acetone and cyclohexane. The VOCs 2-methylbutane (*p* = 0.038) and acetone (*p* = 0.002) were significantly decreased and cyclohexane was significantly increased (*p* = 0.004) in CDH patients compared to the healthy group (Figure 1).

### 2.3. 16S Based Airway Microbiome

**Figure 2:**
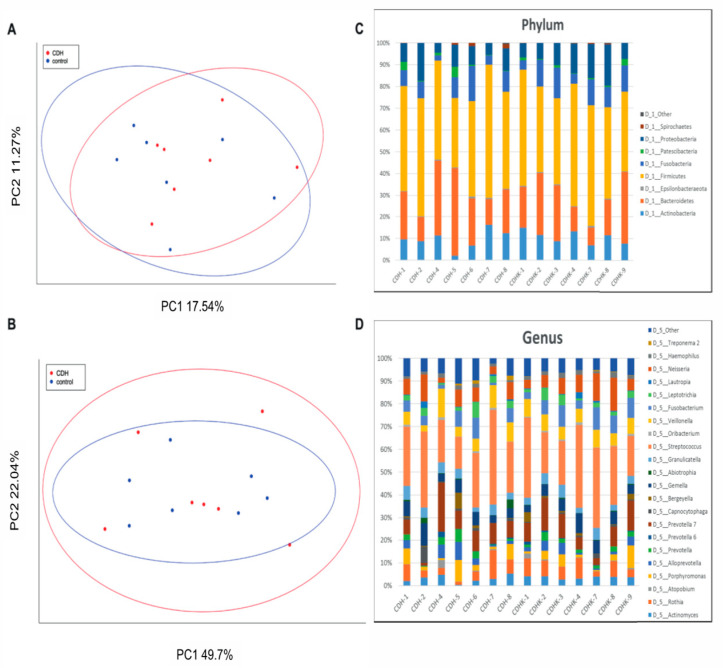
Principial coordinate analysis (PCoA) with weighted UniFrac comparison (**A**) and Bray–Curtis dissimilarity (**B**) tests; 95% confidence ellipses are indicated. The results revealed no obvious clustering of the14 deep induced sputum samples (CDH patients red dots, controls blue dots). PERMANOVA revealed no significant differences in both tests. Relative abundances of CDH patients and age and sex-matched controls at the phylum level (**C**) and genus level (**D**). Note that only bacteria with relative abundances of more than 1% are depicted.

The airway microbiome was measured with 16S based analysis of deep induced sputum samples. Alpha-diversity of the deep induced sputum samples did not differ significantly between CDH patients and controls (Shannon Index CDH median 6.94 ± IQR 0.577 vs. controls median 6.86 ± IQR 0.414; *p* = 0.655). Likewise, LefSe analysis over all hierarchical levels between the two groups did not result in significant different taxa. Beta-diversity analysis was not significantly different between the two groups (weighted unifrac *p* = 0.97, Bray–Curtis *p* = 0.88) (Figure 2). Analysis of the relative abundances revealed no statistically significant differences at the phylum, class and genus level between the two groups studied. On the order and family level, however, the relative abundances of *Pasteurellales* (controls median 0.022 ± IQR 0.01 vs. CDH median 0.016 ± IQR 0.01; *p* = 0.038) and *Pasteurellaceae* (controls median 0.022 ± IQR 0.01 vs. CDH median 0.016 ± IQR 0.01; *p* = 0.038) were significantly lower in CDH patients.

### 2.4. Spirometry

**Figure 3:**
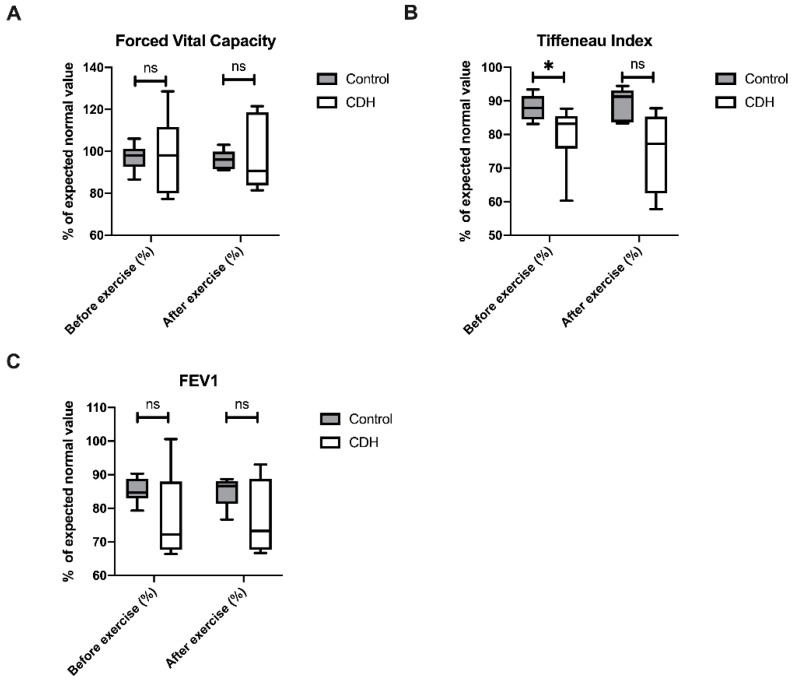
Forced vital capacity (FVC) (**A**), Tiffeneau Index (**B**) and forced expiratory volume in 1 s (FEV1) (**C**) before and after exercise of control patients and CDH patients; ns: not significant; * *p* < 0.05.

Conventional spirometry was performed before and after exercise testing. CDH patients showed no differences in their forced vital capacity (FVC) before and after exercise in comparison to their healthy peers (Figure 3). The Tiffeneau index was significantly lower in CDH patients before (*p* = 0.028), but not after exercise (*p* = 0.063).

### 2.5. Spiroergometry

Bicycle spiroergometry was performed with a sex and body weight dependent protocol. There was no statistically significant difference between patients with CDH and their healthy peers (Table 3).

## 3. Discussion

Our study gives a first insight into both the airway microbiome and volatile organic compounds in breath samples of patients 6 to 13 years following surgical CDH repair compared to healthy age and sex-matched peers. As a major result, it revealed no significant differences in the bacterial airway colonization but differences in the VOC profile.

We were not able to find statistically significant differences regarding anthropometric parameters such as height, body weight, BMI, body fat and muscle mass between the two groups. This is concordant with the literature describing no evidence for long-term growth impairment in patients following CDH repair [15].

Regarding the VOC profiles of exhaled breath samples, CDH patients exhibited significantly decreased levels of acetone and 2-methylbutane, in addition to significantly increased levels of cyclohexane. Acetone is formed by decarboxylation of acetoacetate generated by beta-oxidation of fatty acids and is thus linked to fat metabolism [22]. Under exercise, acetone levels have been shown to increase to the lactate threshold at about 45% of maximum exercise followed by a steady decrease. In this regard the acetone peak marks the switch between fat and carbohydrate metabolism [22]. Type I diabetes and fasting are medical conditions with increased ketone concentrations in breath and urine. Both are associated with predominant lipid metabolism. In type I diabetes, a lack of insulin prevents dextrose from entering the cells leading to impaired carbohydrate metabolism. In this condition, the body shifts to lipid oxidation as the energy source, resulting in increased ketone levels. Similarly, lipid oxidation is activated in response to a lack of carbohydrates under fasting conditions. In our collective, no patient was known to be diabetic. All patients fasted for 2 h prior to VOC sampling. Therefore, the conditions were similar in both studied groups. Since no metabolic parameters were determined in this study, possible unknown co-morbidities cannot be ruled out as reasons for the different acetone levels, especially as there are no known influences of CDH on carbohydrate or fat metabolism. There is currently no scientific information about the role of 2-methylbutane in in vivo experiments. The exact role of this VOC has to be elucidated in future studies. Cyclohexane is an organic solvent and part of raw oil. None of our participants reported increased exposure to organic solvents or gasoline vapors. The distribution of patients exposed to passive smoke did not differ between the groups (compare Table 1). There are no reports focusing on its origin in humans at present. However, cyclohexane has been mentioned in two studies in association with pulmonary diseases. First, Oguma et al. described increased levels of cyclohexane (and xylene) in patients with lung cancer compared to healthy controls (after ruling out possible confounders such as age, smoking status, gender and pulmonary function) [23]. Furthermore, the authors described an increase in cyclohexane (and xylene) levels in breath samples with progressing disease and a decrease in the healing process [23]. A second study revealed increased cyclohexane levels (among other VOCs) in cell cultures of human lung tissues infected with *E. coli*, *P. aeruginosa* and *S. aureus* [24]. Similarly, the authors could demonstrate increased cyclohexane levels in rabbits with pneumonia due to infection with the same pathogens [24]. While xylene showed no significant alterations in our study, cyclohexane was significantly increased as a possible sign for pulmonary impairment in CDH patients.

The airway microbiome analysis showed no significant differences of both α- and β-diversity between CDH and control patients. Regarding patient history, only two of the nine CDH patients reported recurrent respiratory infections. This low number makes a statistical comparison unfeasible. Additionally, the infections occurred before enrollment in this trial and it was therefore impossible to assess the nature of these infections (viral/bacterial). Taken together, the role of the airway microbiome and its role in a possible pre-disposition for respiratory infections remains unclear at present. Currently, there are no other reports in the literature, making comparisons to other patient groups impossible.

Spirometry revealed no significant differences in FVC between CDH and control patients. Similarly, Turchetta and coworkers reported no significant differences in lung function testing in CDH patients [25]. In contrast, however, Zaccara et al. and Marven et al. both have described a significant FVC reduction in CDH patients compared to healthy controls [15,26]. Regarding airway obstruction, our data confirm the findings of other authors who also reported a reduced FEV1 or FEV1/FVC in CDH patients [5,13,26].

While some authors have shown that CDH patients feel less fit than their healthy peers [15,25], the majority of our patients felt physically fit. The bicycle spiroergometry results underline this subjective impression showing no significant differences between CDH and control patients. While Marven and colleagues reported no significant impairment of exercise performance in CDH patients similar to our data [15], other authors have revealed evidence for reduced exercise performance (lower VO_2_max, lower O_2_-pulse or endurance time) in their CDH groups [5,13,14,26]. A possible reason for the discrepancies in this regard may be differences in the training status of the patients. Several studies could prove that CDH patients who exercised had a better performance in spiroergometry compared to those who did not [5,25]. Therefore, a different training status between the CDH and control group may explain some of the differences found in exercise performance testing. While we did not assess the training habits in our study, our collective showed no differences in BMI, muscle or fat mass as possible anthropometric signs influencing the exercise performance between the groups.

Study limitations include that, despite a high effort with repeated attempts to contact patients, the number of recruited patients is relatively low. This can be explained by the low prevalence of CDH of 2.6 out of 10,000 total births and a mortality rate of 37.7% [27] and the single-center setting of this investigation. A further fact decreasing the number of potential participants is the fact that we only recruited children between 6 and 16 years of age in order to assess changes of the examined parameters in children and adolescents. Including older patients might increase the number of confounders (smoking, exhaust, comorbidities, etc.) for the values investigated. Younger children, on the other side, would not have been able to sufficiently participate in controlled breath VOCs sampling. In case of low effect size, relevant group differences which would have been detected in a larger sample size might have been overlooked due to the low number of participants in this study. Therefore, our study can be interpreted as an observational pilot study. Nevertheless, we give a first thorough overview of the airway microbiome and VOC analysis of CDH patients. Moreover, all but one patient underwent direct closure of the diaphragmatic defect. As a potential consequence our patient group may present with better exercise performance and other parameters compared to patients with larger defects and possibly associated higher grad of pulmonary hypoplasia. Therefore, future multi-center trials including a larger group of patients will be required to expand this first data set. Another limitation is that when sampling deep induced sputum, the sample from the deep airways also passes the main bronchi, trachea, pharynx and mouth resulting in a possible contamination of the sample at these levels, probably masking potential biological differences in the deep airways. Therefore, the microbiome sample obtained can only be referred to as an airway microbiome. Harvesting the pulmonary microbiome is only possible by bronchoscopy with a broncho-alveolar lavage, which is ethically impossible in our setting. Further, analyzing the fungal airway microbiome might remain of potential interest. Regarding VOC measurements, effects of inhaled room air could be addressed by sampling room air at each measurement. We consistently excluded potential marker candidates with high room air concentrations in this study. However, there are many factors with possible influences on the breath VOC profile [28,29]. Despite a careful study design and patient questioning, influences of other factors (unknown co-morbidities, influences of diet, etc.) cannot be ruled out completely. In particular, acetone is known to be influenced by patient metabolism. As we did not expect differences in acetone levels, metabolic markers (urine ketone levels, blood dextrose levels or HbA1c) were not determined. Therefore, the reason for the different acetone levels remains unclear at present. Future studies in the CDH collective will have to include metabolic markers to assess the influence of CDH per se on this parameter.

## 4. Materials and Methods

After ethical approval (EK 28-528 ex 15/16) all patients who had undergone surgical CDH repair at our institution were contacted by letter and telephone and invited to participate in this investigation. For all patients, age and sex- matched healthy controls were recruited from families of the medical staff. In all cases written informed consent was obtained from patients and/or legal guardians. We excluded patients younger than 6 years due to potential difficulties with controlled breath gas sampling and exercise and lung function testing. Moreover, patients older than 16 years and children with acute (within 4 weeks before the examination) and chronic gastro-intestinal disease, acute urinary tract infection, antibiotic or probiotic treatment within 4 weeks before the examination were excluded. After inclusion, patients were invited to participate in the following examinations:

### 4.1. Clinical Examination

The clinical examination included investigation of the following anthropometric data: height, body weight (BW) and body mass index (BMI). The body fat in % was determined by the caliper method, as previously described [30]. Appendicular muscle mass was assessed by segmental multi-frequency impedance analysis as published before [31].

### 4.2. Breath VOC Sampling

Patients fasted 2 h before sampling. Alveolar breath gas sampling was performed by combining mainstream capnometry and needle trap microextraction (NTME) with an automated sampling device (PAS Technology Deutschland GmbH, Magdala, Germany), as previously reported [19]. Needle trap devices (NTDs) were pre-conditioned in a heating device (PAS Technology Deutschland GmbH, Magdala, Germany) at 200 °C for 30 min under permanent N_2_-flow before each measurement. This device ensured alveolar sampling at a flow rate of 30 mL/min by means of a CO_2_-triggered, fast responding valve with a CO_2_ threshold of 25–30 mmHg. Sampling was repeated twice for every patient. Additionally, ambient room air was harvested after each patient measurement by automated NTME sampling. After sampling, NTDs were sealed by a Teflon cap (Shinawa LTD. Japan/PAS Technology Deutschland GmbH, Magdala, Germany) immediately. Specimens were measured within 48 h after sampling.

### 4.3. Breath VOC Analysis

An Agilent 7890A gas chromatograph connected to an Agilent 5975 inert XL mass selective detector (MSD) was used for GC-MS measurements, as previously described [19]. A total of 67 substances were identified by means of a mass spectral library (NIST 2005, Gatesburg, PA, USA). The total responses for each substance were recorded. A total of 32 potential marker candidate compounds were verified by pure reference substances. For that purpose, a mixture of gaseous standards (Gas-MIX, Ionicon Analytik GmbH, Innsbruck, Austria) and aqueous solutions of pure reference substances (Sigma Aldrich, Darmstadt, Germany) were evaporated by means of a liquid calibration unit (LCU, Ionicon Analytik GmbH, Innsbruck, Austria). Concentration levels of the gas standards were prepared from 1 ppb to 500 ppb by diluting the standards with nitrogen and water with a matrix adapted humidity of 25 g/m^3^ as previously described [32,33]. Evaporated standard gas was pre-concentrated onto NTDs and analyzed by GC-MS.

For the calibration and determination of limit of detection (LOD, signal-to-noise ratio 3:1) and limit of quantification (LOQ, signal-to-noise ratio 10:1), different concentration levels of the reference substances were measured as previously described [34] (Supplementary Table S1). The signals of selected ions from the reference substances at different concentration levels were used to calculate a calibration curve for each substance. These curves allowed to derive the concentrations of marker substances in parts per billion per volume (ppbV). The median VOC concentrations for patients´ and room air derived substances were compared. If the room air concentration of a candidate substance exceeded 20% of the patients´ median the observed changes were considered as biased by room air contamination and therefore excluded as potential marker compounds.

### 4.4. 16S Based Airway Microbiome

Deep induced sputum samples were harvested as previously described in the literature [35]. Samples were stored at −80 °C until further measurement. Briefly, sputum samples were treated with 100 µg/mL DTT (Sigma), incubated at 37 °C for 20 min and centrifuged at 4000× *g* for 30 min. Supernatant was removed and the pellet was resuspended in 500 µL PBS (Roth). A total of 250 µL of the suspension were mixed with 250 µL bacterial lysis buffer (Roche, Mannheim, Germany) and total DNA was isolated according to manufacturer’s instructions and as published [36] in a MagNA Pure LC 2.0 (Roche, Mannheim, Germany) with the MagNA Pure LC DNA Isolation Kit III (Bacteria, Fungi) (Roche, Mannheim, Germany). A total of 5 µL of total DNA was used in a 25 µL PCR reaction in triplicates using a Fast Start High Fidelity PCR system (Roche, Mannheim, Germany) according to Klymiuk et al. [36], with the target specific primers F27-AGAGTTTGATCCTGGCTCAG and R357-CTGCTGCCTYCCGTA [37]. The 6 pM library was sequenced on an Illumina MiSeq desktop sequencer (Eindhoven, The Netherlands) with 20% PhiX control DNA (Illumina, Eindhoven, The Netherlands) and v3 chemistry for 600 cycles in paired end mode according to manufacturer’s instructions and FastQ raw reads were used for data analysis. A total of 2,711,449 (per sample minimum 94,894, maximum 235,181, mean 193,674) raw sequence reads were used for data analysis in an established Galaxy based workflow (Medical University of Graz, funded by the Austrian Federal Ministry of Education, Science and Research, Hochschulraum-Strukturmittel 2016 grant as part of BioTechMed Graz). Briefly, raw reads were quality-filtered, de-noised, de-replicated, merged and checked for chimeras using DADA2 pipeline [38] with standard settings in QIIME2.0 [39]. For taxonomic assignment SILVA rRNA database Release 132 at 97% identity was used.

### 4.5. Spirometry

Spirometry was performed before and after exercise testing (Oxycon Pro^®^, Carl Reiner GmbH, Vienna, Austria). Forced vital capacity (FVC) was assessed as the maximum amount of air exhaled after maximum inhalation and expressed as percent predicted values. Forced expiratory volume in 1 s (FEV1) was determined and used to calculate the Tiffeneau index (FEV1/FVC).

### 4.6. Spiroergometry

Bicycle spiroergometry (Excalibur Sport^®^, Lode B.V., Groningen, The Netherlands and spirometer Oxycon Pro^®^, Carl Reiner GmbH, Vienna, Austria) was performed with a sex and body weight dependent protocol [30]. The spiroergometry was continued to exhaustion followed by a 3-min recovery phase. The respiratory parameters included tidal volume, respiratory rate, minute volume (MV) and inspiratory (FiO_2_) and expiratory (FeO_2_) fraction of oxygen. The accuracy for FiO_2_ and FeO_2_ is given with ±0.01 vol% by the manufacturer. Using these values and the minute volume the oxygen uptake was calculated. For each patient we recorded the maximum performance per kilogram body weight, the maximum oxygen uptake per kilogram body weight, the relative performance capacity, the respiratory exchange ratio and the oxygen pulse.

### 4.7. Statistics

All data were managed with Microsoft Excel 2016^®^ (Microsoft Corporation, Redmond, WA, USA). For statistical analysis, data were transferred to SPSS 25.0^®^ (IBM Austria, Vienna, Austria). Metric data are displayed as median (interquartile range, IQR). A Mann–Whitney U-Test was performed for group comparison. *p*-values <0.05 were regarded as statistically significant. Box plots were drawn with Prism 8.3.0^®^ (GraphPad, San Diego, CA, USA) and heatmaps with R Studio 1.2.1335^®^ (RStudio Inc., Boston, MA, USA) using the heatmap.2 library.

## 5. Conclusions

In conclusion, this is the first study to report on the airway microbiome and VOC profile in CDH. The alterations of the microbiome were minor and the clinical consequence of reduced Pasteurellaceae remains unclear at present. The elevations in cyclohexane levels that were observed in the CDH group have also been reported in cases of lung cancer and pneumonia. CDH patients showed signs of an obstructive pulmonary disease. CDH patients had no signs of impaired physical performance capacity mirroring controversial reports in the literature in this regard. Future larger scale multi-center studies will be required to confirm these first results.

## Figures and Tables

**Table 1:** Overview of CDH patients and their age and sex-matched healthy controls.

CDH	Control	Age	Gender	Muscle Mass	Body Fat	VOCs	Pulmonary Microbiome	Spirometry Before Ex.	Spiroergometry	Spirometry After Ex.
CDH-1	CDHK-7	12	m	X	X	X	X	X	X	X
CDH-2	CDHK-8	9	m	X	X	X	X	X	01	01
CDH-3	CDHK-6	8	m	X	X	X	01	X	01	01
CDH-4	CDHK-9	13	m	X	X	X	X	X	X	X
CDH-5	CDHK-2	9	f	X	X	X	X	X	X	X
CDH-6	CDHK-4	12	f	X	X	X	X	X	X	X
CDH-7	CDHK-3	6	m	01	X	X	X	X	01	01
CDH-8	CDHK-1	13	m	X	X	X	X	X	X	X
CDH-9	CDHK-5	7	f	02	02	X	02	02	02	02

X: Examination performed and valid; 0: Examination of one or both of the matched pairs missing; Ex: Exercise; Underlined: Patients have been subjected to passive smoke in their familial surroundings; 1: Patient physically unable to perform test/donate sample; 2: Patient refused to perform test/donate sample. CDH: congenital diaphragmatic hernia; VOC: volatile organic compound.

**Table 2:** Anthropometric data of the patients in the CDH and control group. Data presented as medians (IQRs) and the Mann–Whitney U test was performed for group comparison.

Parameter	Control Group	CDH Group	*p*-Value
Height (cm)	137.0 (39.5)	142.5 (36.5)	0.673
Body weight (kg)	30.0 (28.4)	36.4 (30.5)	0.673
BMI (kg/m^2^)	16.0 (5.0)	18.0 (4.7)	0.888
Appendicular muscle mass (kg/m^2^)	5.6 (3.6)	5.3 (2.8)	0.805
Body fat (%)	5.3 (2.8)	6.0 (15.0)	0.442

BMI: Body Mass Index.

**Table 3:** Results of exercise performance testing (exhausting bicycle spiroergometry). Data displayed as medians (IQRs).

Parameter	Control Group	CDH Group	*p*-Value
Relative Performance Capacity (%)	118.0 (27.0)	108.0 (33.0)	0.095
VO_2_max/kg (mL/kg/min)	46.7 (12.3)	42.3 (9.6)	0.222
Pmax/kg (W/kg)	3.4 (1.0)	3.3 (0.8)	0.310
O_2_ Pulse (mL)	12.1 (7.3)	10.2 (7.6)	1.0
RER	1.2 (0.2)	1.2 (0.1)	0.841

VO_2_max/kg: Maximum oxygen uptake per kilogram body weight; Pmax/kg: Maximum performance per kilogram body weight; RER: Respiratory exchange ratio.

## Data Availability

The data presented in this study are available on request from the corresponding author.

## Supplementary Materials

The following are available online, Figure S1: Exhaled and ambient room air concentrations (ARA) of VOC candidate substances regarded as affected by room air; Figure S2: Exhaled and ambient room air concentrations (ARA) of VOC candidate substance without significant differences between CDH patients and controls; Table S1: Limit of detection (LOD) and limit of quantification (LOQ) of 32 candidate VOCs detected in breath samples by needle trap micro-extraction (NTME).

## Registration

This trial was registered with www.clinicaltrials.gov (NCT 03787160).

## Sample Availability

Samples of the compounds are not available from the authors.

## References

[1] Flemmer A.W., Jani J.C., Bergmann F., Muensterer O.J., Gallot D., Hajek K., Sugawara J., Till H., Deprest J. (2007). Lung tissue mechanics predict lung hypoplasia in a rabbit model for congenital diaphragmatic hernia. Pediatr. Pulmonol..

[2] Zalla J.M., Stoddard G.J., Yoder B.A. (2015). Improved mortality rate for congenital diaphragmatic hernia in the modern era of management: 15year experience in a single institution. J. Pediatr. Surg..

[3] Skari H., Bjornland K., Haugen G., Egeland T., Emblem R. (2000). Congenital diaphragmatic hernia: A meta-analysis of mortality factors. J. Pediatr. Surg..

[4] Bohn D. (2002). Congenital Diaphragmatic Hernia. Am. J. Respir. Crit. Care Med..

[5] Bojanić K., Grizelj R., Dilber D., Saric D., Vuković J., Pianosi P.T., Driscoll D.J., Weingarten T.N., Pritišanac E., Schroeder D.R. (2016). Cardiopulmonary exercise performance is reduced in congenital diaphragmatic hernia survivors. Pediatr. Pulmonol..

[6] Bagolan P., Casaccia G., Crescenzi F., Nahom A., Trucchi A., Giorlandino C. (2004). Impact of a current treatment protocol on outcome of high-risk congenital diaphragmatic hernia. J. Pediatr. Surg..

[7] Peetsold M.G., Heij H.A., Nagelkerke A.F., Ijsselstijn H., Tibboel D., Quanjer P.H., Gemke R.J.B.J. (2009). Pulmonary function and exercise capacity in survivors of congenital diaphragmatic hernia. Eur. Respir. J..

[8] Trachsel D., Selvadurai H., Bohn D., Langer J.C., Coates A.L. (2005). Long-term pulmonary morbidity in survivors of congenital diaphragmatic hernia. Pediatr. Pulmonol..

[9] Basek P., Bajrami S., Straub D., Moeller A., Baenziger O., Wildhaber J., Bernet V. (2008). The pulmonary outcome of long-term survivors after congenital diaphragmatic hernia repair. Swiss Med Wkly..

[10] Brar T., Nagaraj S., Mohapatra S. (2012). Microbes and asthma. Curr. Opin. Pulm. Med..

[11] Boutin S., Graeber S.Y., Weitnauer M., Panitz J., Stahl M., Clausznitzer D., Kaderali L., Einarsson G., Tunney M.M., Elborn J.S. (2015). Comparison of Microbiomes from Different Niches of Upper and Lower Airways in Children and Adolescents with Cystic Fibrosis. PLoS ONE.

[12] Morris A., Beck J.M., Schloss P.D., Campbell T.B., Crothers K., Curtis J.L., Flores S.C., Fontenot A.P., Ghedin E., Huang L. (2013). Comparison of the respiratory microbiome in healthy nonsmokers and smokers. Am. J. Respir. Crit. Care Med..

[13] Gischler S.J., van der Cammen-van Zijp M.H.M., Mazer P., Madern G.C., Bax N.M., De Jongste J.C., Van Dijk M., Tibboel D., Ijsselstijn H. (2009). A prospective comparative evaluation of persistent respiratory morbidity in esophageal atresia and congenital diaphragmatic hernia survivors. J. Pediatr. Surg..

[14] van der Cammen-van Zijp M.H.M., Gischler S.J., Mazer P., Van Dijk M., Tibboel D., Ijsselstijn H. (2010). Motor-function and exercise capacity in children with major anatomical congenital anomalies: An evaluation at 5years of age. Early Hum. Dev..

[15] Marven S.S., Smith C.M., Claxton D., Chapman J., Davies H.A., Primhak R.A., Powell C.V.E. (1998). Pulmonary function, exercise performance, and growth in survivors of congenital diaphragmatic hernia. Arch. Dis. Child..

[16] Miekisch W., Schubert J., Nöldge-Schomburg G. (2004). Diagnostic potential of breath analysis—focus on volatile organic compounds. Clin. Chim. Acta.

[17] Van De Kant K.D., Van Der Sande L.J.T.M., Jöbsis Q., Van Schayck O.C.P., Dompeling E. (2012). Clinical use of exhaled volatile organic compounds in pulmonary diseases: A systematic review. Respir. Res..

[18] Trefz P., Obermeier J., Lehbrink R., Schubert J.K., Miekisch W., Fischer D.-C. (2019). Exhaled volatile substances in children suffering from type 1 diabetes mellitus: Results from a cross-sectional study. Sci. Rep..

[19] Trefz P., Rösner L., Hein D., Schubert J.K., Miekisch W. (2013). Evaluation of needle trap micro-extraction and automatic alveolar sampling for point-of-care breath analysis. Anal. Bioanal. Chem..

[20] Obermeier J., Trefz P., Happ J., Schubert J.K., Staude H., Fischer D.-C., Miekisch W. (2017). Exhaled volatile substances mirror clinical conditions in pediatric chronic kidney disease. PLoS ONE.

[21] Barker M., Hengst M., Schmid J., Buers H.-J., Mittermaier B., Klemp D., Koppmann R. (2006). Volatile organic compounds in the exhaled breath of young patients with cystic fibrosis. Eur. Respir. J..

[22] Schubert R., Schwoebel H., Mau-Moeller A., Behrens M., Fuchs P., Sklorz M., Schubert J.K., Bruhn S., Miekisch W. (2012). Metabolic monitoring and assessment of anaerobic threshold by means of breath biomarkers. Metabolomics.

[23] Oguma T., Nagaoka T., Kurahashi M., Kobayashi N., Yamamori S., Tsuji C., Takiguchi H., Niimi K., Tomomatsu H., Tomomatsu K. (2017). Clinical contributions of exhaled volatile organic compounds in the diagnosis of lung cancer. PLoS ONE.

[24] Zhou Y., Chen E., Wu X., Hu Y., Ge H., Xu P., Zou Y., Jin J., Wang P., Ying K. (2017). Rational lung tissue and animal models for rapid breath tests to determine pneumonia and pathogens. Am. J. Transl. Res..

[25] Turchetta A., Fintini D., Cafiero G., Calzolari A., Giordano U., Cutrera R., Morini F., Braguglia A., Bagolan P. (2011). Physical activity, fitness, and dyspnea perception in children with congenital diaphragmatic hernia. Pediatr. Pulmonol..

[26] Zaccara A., Turchetta A., Calzolari A., Iacobelli B., Nahom A., Lucchetti M., Bagolan P., Rivosecchi M., Coran A. (1996). Maximal oxygen consumption and stress performance in children operated on for congenital diaphragmatic hernia. J. Pediatr. Surg..

[27] Politis M.D., Bermejo-Sánchez E., Canfield M.A., Contiero P., Cragan J.D., Dastgiri S., De Walle H.E., Feldkamp M.L., Nance A., Groisman B. (2020). Prevalence and mortality in children with congenital diaphragmatic hernia: A multicountry study. Ann. Epidemiol..

[28] Fischer S., Bergmann A., Steffens M., Trefz P., Ziller M., Miekisch W., Schubert J.S., Köhler H., Reinhold P. (2015). Impact of food intake on in vivo VOC concentrations in exhaled breath assessed in a caprine animal model. J. Breath Res..

[29] Fischer S., Trefz P., Bergmann A., Steffens M., Ziller M., Miekisch W., Schubert J.S., Köhler H., Reinhold P. (2015). Physiological variability in volatile organic compounds (VOCs) in exhaled breath and released from faeces due to nutrition and somatic growth in a standardized caprine animal model. J. Breath Res..

[30] Windhaber J., Steinbauer M., Castellani C., Singer G., Till H., Schober P. (2019). Do Anthropometric and Aerobic Parameters Predict a Professional Career for Adolescent Skiers?. Int. J. Sports Med..

[31] Skrabal F., Pichler G.P., Penatzer M., Steinbichl J., Hanserl A.-K., Leis A., Loibner H. (2017). The Combyn™ ECG: Adding haemodynamic and fluid leads for the ECG. Part II: Prediction of total body water (TBW), extracellular fluid (ECF), ECF overload, fat mass (FM) and “dry” appendicular muscle mass (AppMM). Med. Eng. Phys..

[32] Oertel P., Bergmann A., Fischer S., Trefz P., Küntzel A., Reinhold P., Köhler H., Schubert J., Miekisch W. (2018). Evaluation of needle trap micro-extraction and solid-phase micro-extraction: Obtaining comprehensive information on volatile emissions from in vitro cultures. Biomed. Chromatogr..

[33] Traxler S., Bischoff A.-C., Saß R., Trefz P., Gierschner P., Brock B., Schwaiger T., Karte C., Blohm U., Schröder C. (2018). VOC breath profile in spontaneously breathing awake swine during Influenza A infection. Sci. Rep..

[34] Trefz P., Koehler H., Klepik K., Möbius P., Reinhold P., Schubert J.K., Miekisch W. (2013). Volatile Emissions from Mycobacterium avium subsp. paratuberculosis Mirror Bacterial Growth and Enable Distinction of Different Strains. PLoS ONE.

[35] Pettigrew M.M., Gent J.F., Kong Y., Wade M., Gansebom S., Bramley A.M., Jain S., Arnold S.L.R., McCullers J.A. (2016). Association of sputum microbiota profiles with severity of community-acquired pneumonia in children. BMC Infect. Dis..

[36] Klymiuk I., Bilgilier C., Stadlmann A., Thannesberger J., Kastner M.-T., Högenauer C., Püspök A., Biowski-Frotz S., Schrutka-Kölbl C., Thallinger G.G. (2017). The Human Gastric Microbiome Is Predicated upon Infection with Helicobacter pylori. Front. Microbiol..

[37] McKenna P., Hoffmann C., Minkah N., Aye P.P., Lackner A., Liu Z., Lozupone C.A., Hamady M., Knight R., Bushman F.D. (2008). The Macaque Gut Microbiome in Health, Lentiviral Infection, and Chronic Enterocolitis. PLoS Pathog..

[38] Callahan B.J., Mcmurdie P.J., Rosen M.J., Han A.W., Johnson A.J.A., Holmes S.P. (2016). DADA2: High-resolution sample inference from Illumina amplicon data. Nat. Methods.

[39] Bolyen E., Rideout J.R., Dillon M.R., Bokulich N.A., Abnet C.C., Al-Ghalith G.A., Alexander H., Alm E.J., Arumugam M., Asnicar F. (2019). Reproducible, interactive, scalable and extensible microbiome data science using QIIME 2. Nat. Biotechnol..

