# Supplementary material for: Volatile Organic Compounds, Bacterial Airway Microbiome, Spirometry and Exercise Performance of Patients after Surgical Repair of Congenital Diaphragmatic Hernia
Source: Molecules. 2021 Jan 26;26(3):645. doi: 10.3390/molecules26030645 (PMC7865878; doi:10.3390/molecules26030645)
Supplement: Supplementary file 1 [file molecules-26-00645-s001.pdf]

FIGURE S1: Exhaled and ambient room air (ARA) concentrations of VOC candidate substances regarded as affected by room air. P-values depicted result from group comparison between CDH and Control (Mann-Whitney-U-Test).

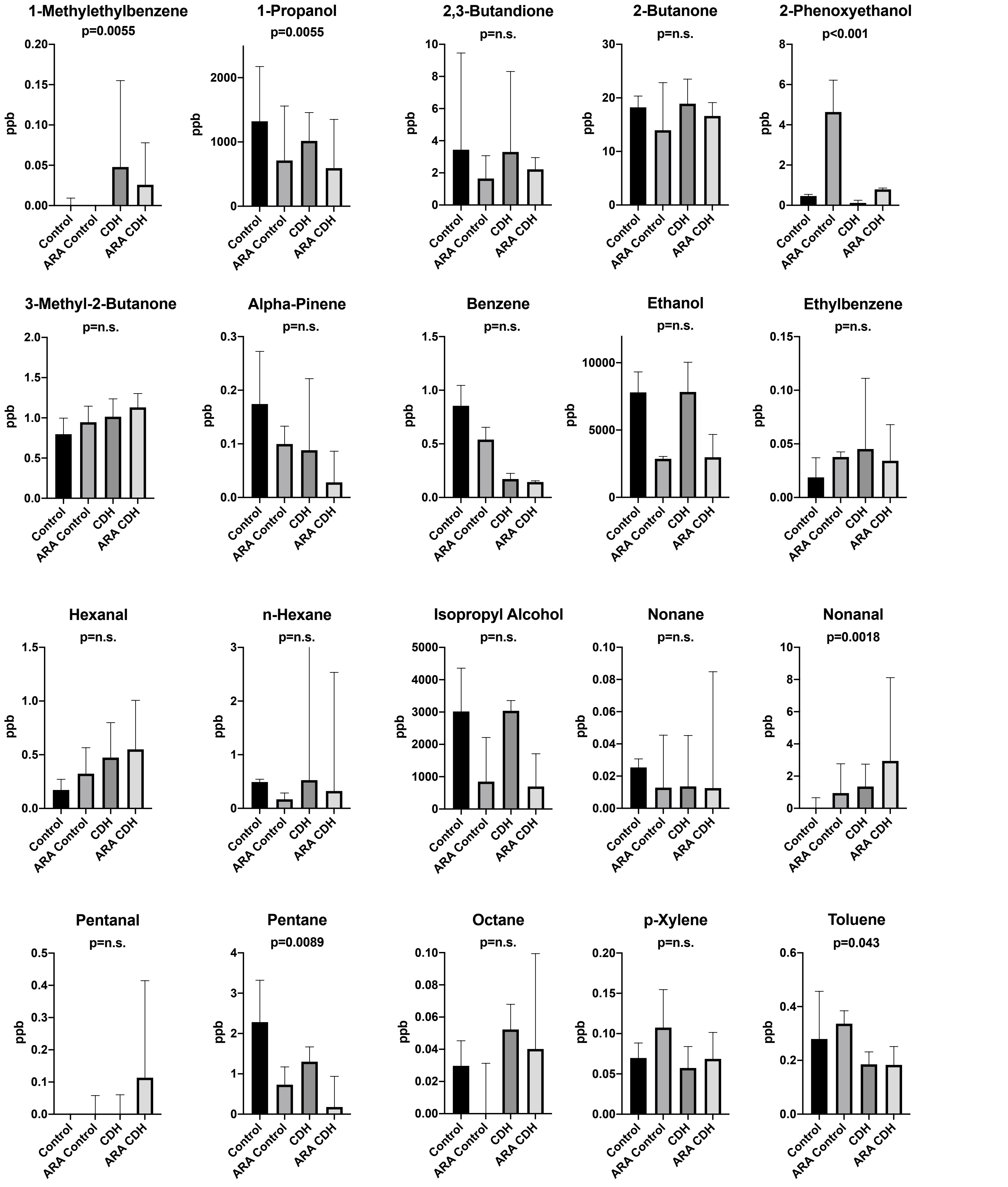

FIGURE S2: Exhaled and ambient room air (ARA) concentrations of VOC candidate substances without significant differences between CDH patients and controls. P-values depicted result from comparison between CDH and Control (Mann-Whitney-U-Test).

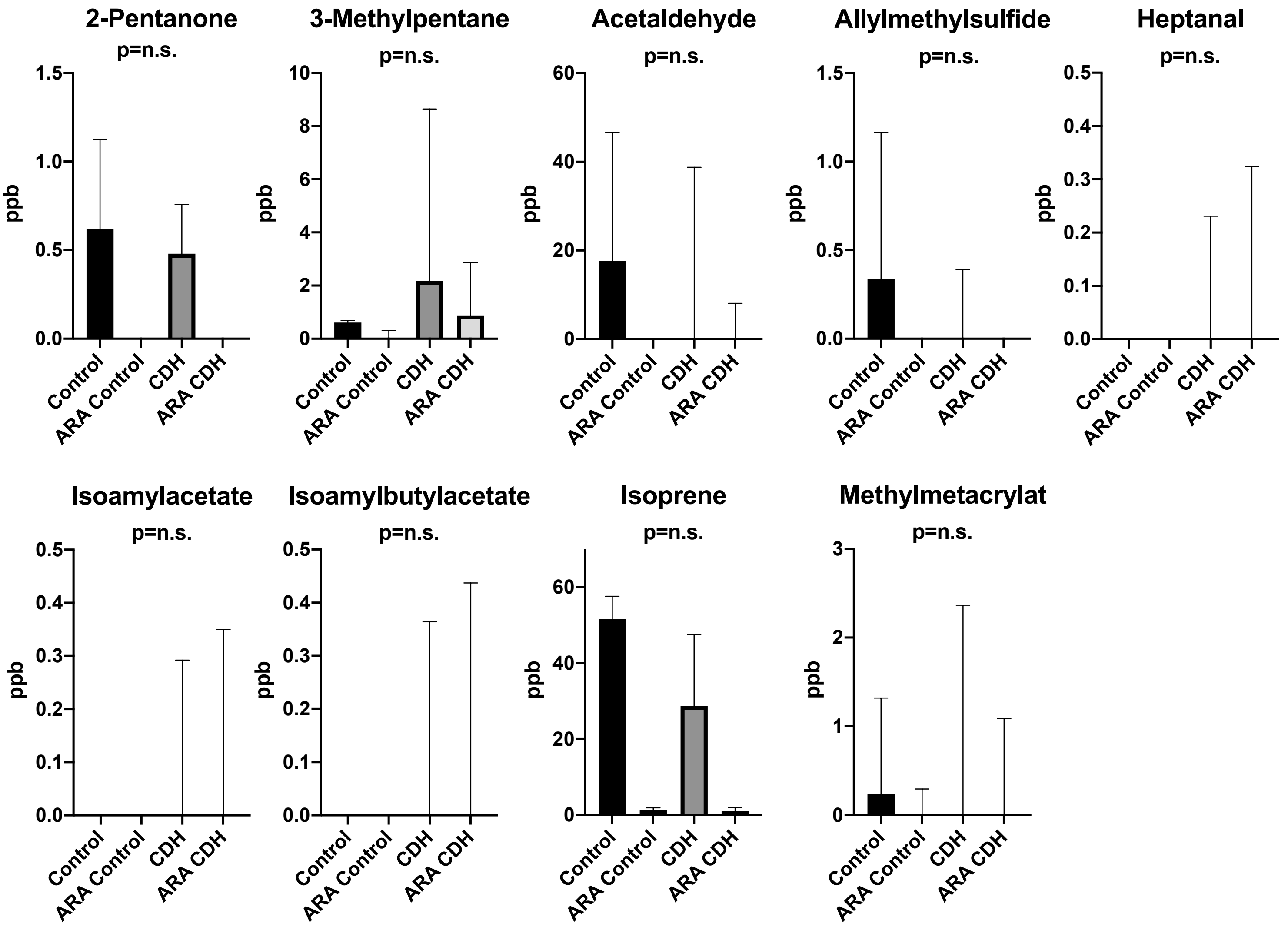

Table S1: Limit of detection (LOD) and limit of quantification (LOQ) of marker substances detected in breath samples by needle trap micro-extraction (NTME).

Substance	LOD	LOQ
1-Methylethylbenzene	0.0039	0.0065
1-Propanol	10.0785	19.7395
2.3-Butandione	0.3944	0.7422
2-Butanone	0.0373	0.0612
2-Methylbutane	25.9322	46.5077
2-Pentanone	0.0432	0.0725
2-Phenoxyethanol	0.0392	0.0389
3-Methyl-2-Butanone	0.0259	0.0464
3-Methylpentane	0.0085	0.0140
Acetaldehyde	15.1731	29.7423
Acetone	0.3493	0.8572
Allylmethylsulfide	0.0378	0.0673
α-Pinene	0.0085	0.0162
Benzene	0.0614	0.1134
Cyclohexane	0.0208	0.0420
Ethanol	1.3327	3.9635
Ethylbenzene	0.0051	0.0089
Heptanal	0.0665	0.1166
Hexanal	0.0828	0.1656
Isoamylacetate	0.0133	0.0281
Isobutylacetate	0.0243	0.0356
Isoprene	0.0264	0.0486
Isopropyl Alcohol	0.2635	0.4305
Methylmetacrylate	0.4317	0.8455
n-Hexane	0.0076	0.0128
Nonanal	0.3079	0.5027
Nonane	0.0120	0.0144
Octane	0.0085	0.0133
Pentanal	0.0985	0.1856
Pentane	0.0640	0.0994
p-Xylene	0.0090	0.0182
Toluene	0.0261	0.0497
